# Trajectory-based associations between a structured acupressure protocol, blood pressure variability, and autonomic balance in patients with primary hypertension

**DOI:** 10.3389/fmed.2026.1899136

**Published:** 2026-09-29

**Authors:** Mayasyanti Dewi Amir, Nunung Liawati, Ady Waluya, Ratu Hidyah Awaliyah

**Affiliations:** 1 Department of Nursing, Institut Citra International Pangkal Pinang, Bangka Belitung Islands, Indonesia; 2 Department of Nursing, STIKES Sukabumi, Sukabumi, Indonesia

**Keywords:** acupressure, ambulatory, autonomic nervous system, blood pressure monitoring, heart rate variability, hypertension, longitudinal studies

## Abstract

**Background:**

Hypertension remains a major global public health problem, with suboptimal rates of blood pressure control despite widespread pharmacological treatment. Beyond mean blood pressure levels, blood pressure variability (BPV) and autonomic nervous system imbalance, reflected by heart rate variability (HRV), have emerged as important predictors of cardiovascular risk. This study aimed to examine trajectory-based associations between a structured acupressure intervention, blood pressure variability, and autonomic balance in patients with primary hypertension.

**Methods:**

Participants received either structured acupressure (12 sessions over 4 weeks) plus usual care or usual care alone. Blood pressure variability was assessed using 24-hour ambulatory monitoring. Autonomic balance was measured via heart rate variability indices. Linear mixed-effects models estimated longitudinal trajectories. Mediation was tested using structural equation modelling.

**Results:**

Compared with controls, the intervention group showed greater reductions in systolic blood pressure variability (*β* = −1.92, 95% CI −2.78 to −1.06, *p* < .001). High-frequency heart rate variability increased (*β* = 84.60, 95% CI 47.60 to 121.60, *p* < .001), and the low/high-frequency ratio decreased (*β* = −0.42, 95% CI −0.60 to −0.24, *p* < .001). Exploratory mediation analysis identified a significant indirect association, suggesting that changes in autonomic balance may partially account for the observed relationship.

**Conclusion:**

Structured acupressure was associated with favorable longitudinal changes in blood pressure variability and autonomic function compared with usual care. Mediation analysis suggested that changes in autonomic balance may partially account for the observed association; however, these findings should be interpreted cautiously because the quasi-experimental design does not allow causal inference. Randomized sham-controlled studies are required to confirm these findings.

## 1 Introduction

Hypertension remains one of the most common and serious noncommunicable diseases worldwide and continues to pose a major challenge to population health and health system sustainability. The World Health Organization (WHO) estimates that approximately 1.28–1.4 billion adults aged 30–79 years are currently living with hypertension globally, with prevalence showing a steady increase, particularly in low- and middle-income countries (1, 2). Although effective antihypertensive medications and international clinical guidelines are widely available, global levels of hypertension awareness, treatment, and control remain far from optimal. As a result, hypertension continues to contribute substantially to the burden of stroke, coronary heart disease, heart failure, and chronic kidney disease (3). These conditions highlight that hypertension should be understood not only as an individual clinical problem but also as a long-term, systemic public health issue that requires innovative, scalable, and complementary management approaches.

Research attention has gradually shifted beyond the traditional focus on average systolic and diastolic blood pressure values. In recent years, blood pressure variability (BPV), including short-term, day-to-day, and visit-to-visit fluctuations has been increasingly recognized as an important and independent predictor of cardiovascular morbidity and mortality, even after accounting for mean blood pressure levels (4, 5). Elevated BPV reflects instability in cardiovascular regulation and has been associated with endothelial dysfunction, arterial stiffness, and target organ damage, which together increase the risk of stroke and myocardial (6, 7). Despite its growing clinical relevance, BPV is still rarely assessed in routine practice and remains insufficiently explored as a primary outcome in nonpharmacological hypertension intervention studies.

One of the key mechanisms underlying increased BPV is autonomic nervous system dysregulation, characterized by excessive sympathetic activity and reduced parasympathetic (vagal) modulation. Heart rate variability (HRV) provides a non-invasive and physiologically meaningful indicator of autonomic balance and has been consistently associated with hypertension severity, resistant hypertension, and cardiovascular prognosis (8, 9). Autonomic imbalance not only contributes to persistent blood pressure elevation but also promotes greater blood pressure instability over time, underscoring the importance of interventions that are able to restore autonomic equilibrium.

Recent evidence has further strengthened the role of autonomic dysfunction in hypertension by demonstrating that reduced heart rate variability is associated not only with impaired blood pressure regulation but also with psychological stress, emotional regulation, and neurocardiac interactions. Emerging studies have reported that autonomic imbalance contributes to cardiovascular instability through complex heart–brain interactions, emphasizing HRV as an important biomarker for cardiovascular risk stratification and therapeutic monitoring (8).

Within this context, acupressure has attracted increasing attention as a complementary and non-invasive intervention derived from traditional Chinese medicine. Acupressure involves manual stimulation of specific acupuncture points to influence neurovascular, neuroendocrine, and autonomic pathways without the use of needles. This feature makes acupressure particularly suitable for nursing practice and patient self-management, especially in community and primary care settings (10). From a physiological perspective, acupressure is believed to support cardiovascular regulation through its effects on autonomic nervous system activity, neurohumoral responses, and vascular function (11).

Growing empirical evidence supports the beneficial role of acupressure in reducing blood pressure among individuals with primary or essential hypertension. Randomized controlled trials have shown that auricular acupressure can significantly improve systolic and diastolic blood pressure, as well as stress and sleep-related outcomes, in adult and older populations with hypertension (12). These findings are further supported by systematic reviews and meta-analyses demonstrating clinically meaningful blood pressure reductions following auricular therapy, particularly when delivered through structured and repeated intervention protocols (13). However, only a limited number of studies have specifically examined the effects of acupressure on autonomic regulation, as measured by HRV indices. Available evidence suggests that acupressure may help reduce sympathetic overactivity and enhance parasympathetic modulation, thereby improving cardiovascular regulation (8). Nevertheless, results across studies remain heterogeneous, likely due to differences in acupoint selection, intervention dose, duration, and outcome measurement methods.

Despite the expanding body of literature, several important gaps remain. First, most acupressure studies continue to focus on pre–post changes in mean blood pressure, while BPV as an outcome with strong clinical and mechanistic relevance has rarely been evaluated as a primary endpoint. Second, autonomic outcomes such as HRV are often treated as secondary or exploratory variables rather than being integrated into a comprehensive mechanistic framework linking the intervention to cardiovascular regulation. Third, many existing studies rely on static comparisons between baseline and endpoint values, without considering that blood pressure and autonomic function are dynamic processes that change over time. Consequently, little is known about the trajectories of BPV and autonomic balance during and after structured acupressure interventions, including whether benefits appear early, increase gradually, or vary across patient subgroups (4, 5). Addressing these gaps is essential to move acupressure beyond a symptom-focused complementary therapy toward a more evidence-informed complementary approach in hypertension management. A trajectory-based analytical approach, which examines longitudinal patterns of BPV and HRV across repeated measurements, provides a valuable framework for capturing dynamic treatment responses, individual variability, and sustained autonomic adaptation. This approach is consistent with current precision health perspectives and responds to the growing call for more rigorous, longitudinal, and mechanism-oriented hypertension research.

Therefore, the present study aims to examine the trajectory-based effects of a structured acupressure intervention on blood pressure variability and autonomic balance in patients with primary hypertension. Specifically, this study seeks to describe longitudinal changes in BPV indices and HRV parameters throughout the intervention period and to compare these trajectories between intervention and control groups. It is hypothesized that patients receiving structured acupressure will demonstrate more favorable BPV trajectories, characterized by reduced variability over time, compared with controls. In addition, structured acupressure is expected to be associated with improved autonomic balance trajectories, reflected by increased parasympathetic activity and reduced sympathetic dominance as measured by HRV indices. Finally, to explore whether longitudinal changes in autonomic balance are statistically associated with changes in blood pressure variability, providing preliminary evidence that warrants confirmation in randomized controlled trials.

## 2 Methods

### 2.1 Study design

This study employed a quasi-experimental repeated measures design to examine the trajectory-based associations between structured acupressure and blood pressure variability and autonomic balance on blood pressure variability and autonomic balance in patients with primary hypertension. A longitudinal framework was used to capture within-individual changes over time and to evaluate dynamic patterns of cardiovascular and autonomic regulation, which are increasingly recognized as clinically meaningful beyond single blood pressure values (14, 15). Randomization was not feasible due to clinical service constraints; therefore, a non-equivalent control group design was applied. Blood pressure variability was assessed at baseline, week 2, week 4, and week 8, whereas heart rate variability was assessed at baseline, week 4, and week 8. The overall study design and timing of the intervention and outcome assessments are illustrated in Figure 1.

**Figure 1:**
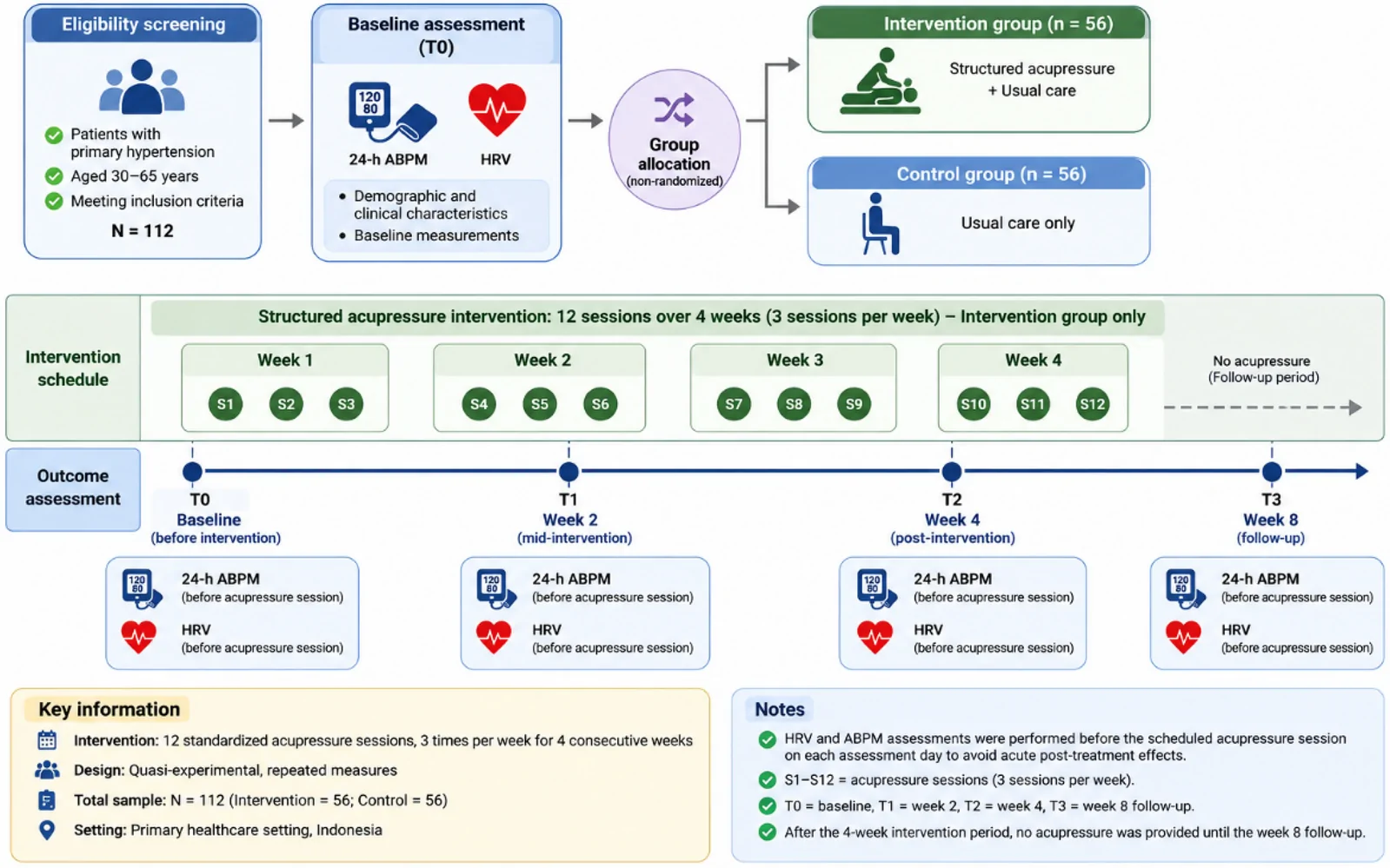
Study design and timeline of the structured acupressure intervention. Eligible participants with primary hypertension underwent baseline assessment (T0) before allocation to either the structured acupressure plus usual care group or the usual care control group. The intervention consisted of 12 standardized acupressure sessions delivered three times per week over four consecutive weeks. Outcome assessments, including 24-hour ambulatory blood pressure monitoring (ABPM) and heart rate variability (HRV), were conducted at baseline (T0), week 2 (T1), week 4 (T2), and week 8 follow-up (T3). On assessment days, HRV and ABPM measurements were performed before the scheduled acupressure session to minimize acute post-treatment effects and better reflect longitudinal physiological adaptation.

### 2.2 Participants

The study was conducted in outpatient hypertension clinics affiliated with a tertiary referral hospital in Indonesia between March and August 2025. The target population consisted of adult patients diagnosed with primary hypertension who were receiving routine outpatient care.

Participants were eligible if they were aged 30–65 years, had a confirmed diagnosis of primary hypertension according to established clinical criteria, had been on a stable antihypertensive medication regimen, to minimize potential confounding, participants were required to maintain stable antihypertensive medication throughout the study period. Medication type and dosage were documented at baseline, and participants were instructed not to modify their treatment unless medically indicated. In addition, participants were advised to avoid excessive caffeine intake, alcohol consumption, vigorous physical activity, and smoking for at least 12 h before HRV assessment to reduce acute physiological influences on autonomic measurements. For at least four weeks prior to enrollment, and were able to communicate in Bahasa Indonesia and provide written informed consent. Participants were excluded if they had secondary hypertension, documented cardiac arrhythmias or heart failure, diabetes mellitus with suspected autonomic neuropathy, neurological disorders affecting autonomic regulation, current use of complementary therapies influencing blood pressure or autonomic function within the previous three months, dermatological or musculoskeletal conditions preventing acupressure application, or if they were pregnant or breastfeeding.

Sample size estimation was performed using G*Power version 3.1.9.7 for repeated measures analysis in a between-group design. A medium effect size of *f* = 0.25 was assumed based on prior studies examining autonomic modulation and blood pressure outcomes (19). With an alpha level of 0.05, statistical power of 0.80, four repeated measurements, and an estimated correlation of 0.50 among repeated measures, the minimum required sample size was 92 participants. To account for an anticipated attrition rate of approximately 20% commonly observed in longitudinal behavioral interventions, a total of 112 participants were recruited.

A consecutive sampling technique was applied. Eligible patients attending the participating clinics during the recruitment period were screened sequentially and invited to participate. Group allocation was determined based on clinic scheduling and service flow to minimize intervention contamination.

### 2.3 Intervention: structured acupressure protocol

The intervention consisted of a structured acupressure protocol designed to target autonomic nervous system modulation. The protocol was developed based on traditional Chinese medicine principles and empirical evidence demonstrating the influence of specific acupoints on autonomic regulation and cardiovascular function (17). Acupressure was delivered by registered nurses who had completed formal training in complementary nursing therapies and received protocol-specific instruction prior to the study.

The protocol targeted bilateral acupoints PC6 (Neiguan), LI11 (Quchi), and ST36 (Zusanli), which have been associated with modulation of sympathetic and parasympathetic activity. Each session lasted approximately 20 min and was administered three times per week over a four-week period, resulting in a total of 12 sessions. Pressure was applied using the thumb in a slow circular motion with moderate and consistent intensity, estimated at 3–5 kg/cm^2^, for two minutes at each acupoint, followed by a one-minute rest interval. Participants were positioned comfortably in a seated or supine posture in a quiet environment to minimize external autonomic stimulation. Intervention fidelity was monitored using standardized session checklists completed by the intervention nurses. Prior to participant recruitment, all intervention nurses completed standardized competency training using a detailed intervention manual. Competency was assessed through supervised practice sessions, and periodic monitoring was performed throughout the study to ensure consistency in acupoint identification, pressure intensity, treatment duration, and protocol adherence.

Participants in the control group received standard hypertension care, including routine pharmacological management and lifestyle counseling, without exposure to acupressure during the study period.

### 2.4 Measurements

Blood pressure variability was assessed using 24-hour ambulatory blood pressure monitoring. Measurements were obtained with a validated oscillometric ambulatory blood pressure monitor calibrated according to manufacturer specifications. Blood pressure readings were recorded at 30-minute intervals during daytime hours and 60-minute intervals during nighttime hours. Blood pressure variability indices included the standard deviation of systolic and diastolic blood pressure and average real variability, calculated from successive readings. Higher values indicated greater blood pressure instabilit (14, 15).

Only recordings with at least 70% of scheduled measurements successfully completed were considered valid for analysis. The ambulatory blood pressure monitor was calibrated according to the manufacturer's recommendations before each use.

Autonomic balance was assessed through heart rate variability analysis using a portable electrocardiographic monitoring device. Five-minute resting ECG recordings were obtained in a quiet, temperature-controlled room with participants in a seated position following a minimum rest period of 10 min. All HRV assessments were performed during the morning (08:00–10:00 a.m.) in a temperature-controlled room under standardized environmental conditions. Participants rested quietly for at least 10 min before recording and were instructed to avoid caffeine, nicotine, alcohol, and strenuous physical activity before assessment. HRV measurements were obtained prior to the scheduled acupressure session to minimize the influence of acute post-treatment relaxation responses.

ECG signals were acquired using a three-lead portable electrocardiographic recorder with an appropriate sampling frequency according to the manufacturer's specifications. Recordings were visually inspected for signal quality, and ectopic beats and recording artifacts were corrected before HRV analysis. HRV parameters were analyzed using validated HRV analysis software following current international recommendations. Participants were instructed to breathe spontaneously without paced respiration throughout the recording.

According to international standards. Frequency-domain parameters included low-frequency power, high-frequency power, and the low-frequency to high-frequency ratio (16). Increased high-frequency power and reduced low-frequency to high-frequency ratio were interpreted as indicators of enhanced parasympathetic activity and improved autonomic balance (17).

The reliability and validity of ambulatory blood pressure monitoring and heart rate variability assessment have been well established in cardiovascular research. Previous studies have reported high reproducibility for ambulatory blood pressure variability indices and intraclass correlation coefficients exceeding 0.85 for short-term heart rate variability measurements under standardized conditions (14).

### 2.5 Procedure

Eligible participants received a detailed explanation of the study objectives, procedures, potential risks, and anticipated benefits. Written informed consent was obtained before enrollment. Baseline data collection was conducted prior to group allocation. Participants in the intervention group then commenced the structured acupressure program, while control participants continued receiving standard care. Outcome measurements were collected at all predefined time points by trained research assistants who were not involved in intervention delivery. At study completion, participants in the intervention group were invited to provide feedback regarding comfort, acceptability, and perceived benefits of the acupressure intervention, which informed feasibility evaluation.

Ambulatory blood pressure monitoring was conducted at baseline, week 2, week 4, and week 8, whereas HRV assessments were performed at baseline, week 4, and week 8.

### 2.6 Data analysis

Data were analyzed using SPSS version 29 and R statistical software. Descriptive statistics were used to summarize demographic and clinical characteristics. Longitudinal changes in blood pressure variability and autonomic balance were examined using linear mixed-effects models with random intercepts to account for within-subject correlations across repeated measurements (18). Fixed effects included group, time, and group-by-time interaction terms to evaluate differential trajectories between intervention and control groups. To explore potential pathways underlying the observed associations, longitudinal mediation analysis was conducted using structural equation modeling. Changes in autonomic balance trajectories were modeled as a mediator of the relationship between structured acupressure and blood pressure variability trajectories (19). Model fit was evaluated using established indices, and statistical significance was set at *p* < .05.

### 2.7 Ethical approval and consent

Ethical approval was obtained from the Institutional Review Board of the affiliated academic institution (Approval No. 0.198.ETIK). All participants provided written informed consent prior to enrolment.

## 3 Results

A total of 112 participants were enrolled at baseline, with 56 assigned to the structured acupressure group and 56 to the control group. Eight participants were lost to follow-up due to scheduling conflicts or withdrawal of consent, resulting in a final analytic sample of 104 participants (52 in each group). Retention rates did not differ between groups.

Baseline demographic and clinical characteristics of participants are presented in Table 1. The mean age of participants was 52.1 ± 8.4 years, and the majority were female. No statistically significant differences were observed between the intervention and control groups with respect to age, sex, body mass index, duration of hypertension, baseline systolic blood pressure, or antihypertensive medication use, indicating baseline comparability (Table 1).

**Table 1:** Baseline demographic and clinical characteristics of participants (*N* = 104).

Characteristic	Intervention (*n* = 52)	Control (*n* = 52)	*p* value
Age (years), mean ± SD	51.8 ± 8.6	52.4 ± 8.2	0.72
Female, *n* (%)	31 (59.6)	30 (57.7)	0.84
Body mass index (kg/m^2^), mean ± SD	25.9 ± 3.4	26.2 ± 3.1	0.63
Duration of hypertension (years), mean ± SD	6.3 ± 3.1	6.6 ± 3.4	0.68
Baseline SBP (mmHg), mean ± SD	142.7 ± 9.8	143.2 ± 10.1	0.79
Baseline DBP (mmHg), mean ± SD	89.6 ± 6.4	90.1 ± 6.1	0.67
Antihypertensive medication use, *n* (%)	52 (100)	52 (100)	–

Table 2 presents longitudinal changes in blood pressure variability and autonomic balance. Baseline values were comparable between groups for systolic blood pressure variability, HF power, and the LF/HF ratio. In the intervention group, systolic blood pressure variability decreased from 13.6 ± 3.2 mmHg at baseline to 9.8 ± 2.4 mmHg at week 8, while the control group remained above 13 mmHg at all time points. The group-by-time interaction was significant (β = −1.92, SE = 0.44, *p* < .001). HF power increased in the intervention group from 312 ± 96 ms^2^ to 468 ± 118 ms^2^, with minimal change in controls (β = 84.6, SE = 18.9, *p* < .001). The LF/HF ratio declined from 2.41 ± 0.62 to 1.59 ± 0.47 in the intervention group, with no meaningful change in controls (β = −0.42, SE = 0.09, *p* < .001) (Table 2).

**Table 2:** Trajectories of blood pressure variability and autonomic balance.

Outcome	Time point	Intervention (*n* = 52) mean ± SD	Control (*n* = 52) mean ± SD
SD of SBP (mmHg)	Baseline (T0)	13.6 ± 3.2	13.4 ± 3.1
	Week 2 (T1)	12.1 ± 2.9	13.2 ± 3.0
	Week 4 (T2)	10.4 ± 2.6	13.1 ± 3.2
	Week 8 (T3)	9.8 ± 2.4	13.0 ± 3.3
HF power (ms^2^)	Baseline (T0)	312 ± 96	318 ± 101
	Week 4 (T2)	426 ± 112	325 ± 98
	Week 8 (T3)	468 ± 118	330 ± 102
LF/HF ratio	Baseline (T0)	2.41 ± 0.62	2.38 ± 0.59
	Week 4 (T2)	1.76 ± 0.51	2.32 ± 0.61
	Week 8 (T3)	1.59 ± 0.47	2.29 ± 0.58

Linear mixed-effects models showed no significant main effect of group for systolic BP variability (β = −0.18, *p* = .56), HF power (β = 6.80, *p* = .58), or LF/HF ratio (β = −0.04, *p* = .62), indicating comparable baseline levels between groups. Significant main effects of time were observed for systolic BP variability (β = −0.64, *p* < .001), HF power (β = 21.30, *p* = .001), and LF/HF ratio (β = −0.09, *p* = .004). Importantly, group-by-time interactions were statistically significant for systolic BP variability (β = −1.92, *p* < .001), HF power (β = 84.60, *p* < .001), and LF/HF ratio (β = −0.42, *p* < .001), demonstrating significantly more favorable trajectories of hemodynamic stability and autonomic balance in the structured acupressure group compared with controls (Table 3).

**Table 3:** Fixed effects estimates from linear mixed-effects models for blood pressure variability and autonomic balance.

Outcome variable	Fixed effect	β	SE	95% CI	*p* value
Systolic BP variability (SD of SBP)	Group (Intervention vs. Control)	−0.18	0.31	−0.79 to 0.43	.56
	Time	−0.64	0.15	−0.94 to −0.35	<.001
	Group×Time	−1.92	0.44	−2.78 to −1.06	<.001
HF power (ms^2^)	Group (Intervention vs. Control)	6.80	12.40	−17.60 to 31.20	.58
	Time	21.30	6.10	9.40 to 33.20	.001
	Group×Time	84.60	18.90	47.60 to 121.60	<.001
LF/HF ratio	Group (Intervention vs. Control)	−0.04	0.08	−0.20 to 0.12	.62
	Time	−0.09	0.03	−0.15 to −0.03	.004
	Group×Time	−0.42	0.09	−0.60 to −0.24	<.001

Longitudinal mediation analysis showed that structured acupressure had a significant positive effect on autonomic balance trajectories (β = 0.52, *p* < .001), which in turn were associated with reductions in blood pressure variability (β = −0.61, *p* < .001). The indirect effect of structured acupressure on blood pressure variability through autonomic balance was statistically significant (β = −0.32, *p* < .001), accounting for 46% of the total effect, while the direct effect remained significant (β = −0.38, *p* = .002), indicating partial mediation (Table 4).

**Table 4:** Longitudinal mediation analysis of autonomic balance in the relationship between structured acupressure and blood pressure variability.

Pathway	Standardized β	SE	95% CI	*p* value
Structured acupressure → autonomic balance (HF power trajectory)	0.52	0.11	0.30 to 0.74	<.001
Autonomic balance → BP variability trajectory	−0.61	0.14	−0.89 to −0.34	<.001
Direct effect: structured acupressure → BP variability	−0.38	0.12	−0.62 to −0.14	.002
Indirect effect via autonomic balance	−0.32	0.09	−0.52 to −0.16	<.001
Total effect	−0.70	–	–	<.001
Proportion mediated	46%	–	–	–

Model fit indices: *χ*^2^/df = 1.87, CFI = 0.96, TLI = 0.95, RMSEA = 0.048.

## 4 Discussion

This study provides longitudinal evidence that structured acupressure was associated with favorable changes in blood pressure variability and autonomic balance among patients with primary hypertension. By applying a repeated measures design and trajectory-based analysis, the findings extend previous acupressure research beyond static blood pressure reduction and demonstrate that structured acupressure influences dynamic cardiovascular regulation over time. Importantly, the observed reductions in systolic blood pressure variability were accompanied by increases in parasympathetic activity and reductions in sympathovagal imbalance, exploratory mediation analysis suggesting that autonomic balance may partially account for the observed association between structured acupressure and blood pressure variability.

Previous clinical studies investigating acupressure in hypertension have primarily focused on changes in systolic and diastolic blood pressure measured at isolated time points. Randomized and quasi-experimental trials have reported modest reductions in blood pressure following acupressure, particularly at points such as PC6 and ST36, but these studies rarely incorporated repeated measurements or mechanistic outcomes related to autonomic function (10). As a result, the extent to which acupressure alters cardiovascular regulation rather than transient blood pressure levels has remained unclear.

The present findings align with and extend prior evidence by demonstrating that acupressure produces sustained changes in autonomic balance, reflected by increased high-frequency heart rate variability and reduced LF/HF ratio. These autonomic shifts are consistent with physiological models describing hypertension as a disorder characterized by chronic sympathetic overactivity and impaired parasympathetic modulation (17). Unlike earlier studies, the present work shows that these autonomic changes follow a clear longitudinal trajectory and precede improvements in blood pressure variability, suggesting a potential regulatory pathway that warrants further investigation.

From a broader cardiovascular perspective, blood pressure variability has emerged as an independent predictor of cardiovascular events and mortality, even among patients with controlled mean blood pressure (14, 15). Contemporary hypertension guidelines increasingly emphasize the importance of out-of-office blood pressure monitoring and longitudinal assessment to better capture hemodynamic instability and cardiovascular risk (3, 20, 21). By demonstrating that structured acupressure reduces blood pressure variability over time, this study contributes novel evidence supporting the role of non-pharmacological interventions in targeting regulatory aspects of hypertension.

Longitudinal mediation analysis suggested that changes in autonomic balance statistically accounted for a substantial proportion of the association between structured acupressure and blood pressure variability. However, given the quasi-experimental design and absence of randomization, these findings should not be interpreted as confirmation of a causal biological pathway. Instead, they provide preliminary evidence supporting further investigation in adequately powered randomized controlled trials.

These findings are consistent with current physiological models in which autonomic nervous system dysregulation plays a causal role in blood pressure instability through altered baroreflex sensitivity, vascular tone, and neurohumoral activation (17). The early emergence of parasympathetic enhancement observed in this study supports the hypothesis that acupressure acts upstream in the regulatory cascade, stabilizing autonomic control before measurable improvements in blood pressure variability occur. Although the temporal sequence observed in this study is consistent with the hypothesis that autonomic modulation may contribute to reductions in blood pressure variability, the quasi-experimental design precludes definitive causal inference. Therefore, the mediation analysis should be interpreted as identifying a potential mechanistic pathway rather than establishing causality. Confirmation of these findings requires randomized controlled trials with longer follow-up periods (18, 19).

Furthermore, placebo effects and participant expectations cannot be completely excluded because the study did not include a sham acupressure control group. The therapeutic interaction, physical touch, and increased attention received during intervention sessions may have contributed to some of the observed physiological responses. Future randomized sham-controlled trials are warranted to better isolate the specific physiological effects of structured acupressure.

### 4.1 Clinical implications

The clinical implications of these findings are substantial. Structured acupressure represents a low-risk, low-cost, and non-invasive intervention that can be delivered by trained nurses as part of routine hypertension management. Unlike many complementary therapies, the structured and protocolized nature of the intervention enhances reproducibility, feasibility, and scalability in real-world clinical settings.

By addressing blood pressure variability and autonomic-related parameters rather than mean blood pressure alone, structured acupressure may have the potential to contribute to cardiovascular risk reduction in patients whose blood pressure appears adequately controlled but who remain at elevated risk due to hemodynamic instability. This is particularly relevant in light of recent guideline updates emphasizing comprehensive blood pressure assessment and individualized management strategies (20, 21). In resource-limited settings, where access to advanced pharmacological or device-based therapies may be constrained, nurse-led acupressure interventions could offer a practical adjunct to standard care.

### 4.2 Study limitations

Several limitations should be acknowledged. First, the quasi-experimental non-randomized design may have introduced selection bias and residual confounding despite comparable baseline characteristics between groups. Second, although antihypertensive medication regimens were stable throughout the study, differences in medication classes and other physiological factors affecting autonomic function, including lifestyle behaviors, could not be completely eliminated. Third, HRV was used as an indirect marker of autonomic regulation, and additional physiological biomarkers such as baroreflex sensitivity or circulating neurohumoral markers would provide a more comprehensive assessment of autonomic function. Fourth, the absence of a sham acupressure control group means that placebo effects and participant expectations cannot be completely excluded. Finally, the single-center setting and relatively short follow-up period may limit the generalizability and long-term interpretation of the findings. Future multicenter randomized controlled trials with longer follow-up periods and broader autonomic assessments are recommended. Although ambulatory blood pressure monitoring was used to derive blood pressure variability indices, mean 24-hour blood pressure values, daytime/nighttime blood pressure, and nocturnal dipping status were not included in the predefined analyses. Future studies should incorporate these parameters to provide a more comprehensive evaluation of the clinical significance of blood pressure variability.

## 5 Conclusion

Structured acupressure was associated with favorable longitudinal changes in blood pressure variability and autonomic function among adults with primary hypertension. Although mediation analysis suggested that autonomic balance may partially account for these associations, the quasi-experimental design and absence of a sham control preclude causal interpretation. Structured acupressure may therefore be considered a promising adjunct to standard hypertension management, but adequately powered randomized sham-controlled multicenter trials with longer follow-up are required before firm clinical conclusions can be drawn.

## Data Availability

The original contributions presented in the study are included in the article/Supplementary Material, further inquiries can be directed to the corresponding author.
